# DioxolaneA3-phosphatidylethanolamines are generated by human platelets and stimulate neutrophil integrin expression

**DOI:** 10.1016/j.redox.2017.01.001

**Published:** 2017-01-13

**Authors:** Maceler Aldrovandi, Christine Hinz, Sarah N. Lauder, Helen Podmore, Martin Hornshaw, David A. Slatter, Victoria J. Tyrrell, Stephen R. Clark, Lawrence J. Marnett, Peter W. Collins, Robert C. Murphy, Valerie B. O’Donnell

**Affiliations:** a Systems Immunity Research Institute, and Institute of Infection and Immunity, School of Medicine, Cardiff University, Cardiff CF14 4XN, UK; b Department of Pharmacology, University of Colorado Denver, Aurora, CO 80045, USA; c ThermoFisher Scientific, Stafford House, Boundary Way, Hemel Hempstead HP2 7GE, United Kingdom; d Vanderbilt Institute of Chemical Biology, Centre in Molecular Toxicology, Vanderbilt-Ingram Cancer Centre, Nashville, TN, USA

**Keywords:** DXA_3_, 8-hydroxy-9, 10-dioxolane A3, PE, phosphatidylethanolamine, COX, cyclooxygenase, PAR, protease-activated receptors, cPLA_2_, cytosolic phospholipase A2, TXA2, thromboxane A2, HETE, hydroxyeicosatetraenoic acid, PGE_2_, prostaglandin E2, OOEPC, oleyloxyethylphosphocholine, BEL, bromoenol lactone, cPLA2i, cytosolic phospholipase A2α inhibitor (N-((2S,4R)-4-(Biphenyl-2-ylmethyl-isobutyl-amino)-1-[2-(2,4-difluorobenzoyl)-benzoyl]-pyrrolidin-2-ylmethyl}-3-[4-(2,4-dioxothiazolidin-5-ylidenemethyl)-phenyl]acrylamide, MAPK, mitogen associated protein kinase, DMPE, dimyristolyphosphatidylethanolamine, AA, arachidonate, SAPE, 1-stearoly-2-arachidonyl-PE, DTPA, diethylenetriaminepentaacetic acid, HCD, higher energy collision-induced-dissociation, MRM, multiple reaction monitoring, LPAT, lysophospholipid acyl transferases, Eicosanoids, Platelets, Cyclooxygenase, Phospholipids, Mass spectrometry

## Abstract

Activated platelets generate an eicosanoid proposed to be 8-hydroxy-9,10-dioxolane A3 (DXA_3_). Herein, we demonstrate that significant amounts of DXA_3_ are rapidly attached to phosphatidylethanolamine (PE) forming four esterified eicosanoids, 16:0p, 18:0p, 18:1p and 18:0a/DXA_3_-PEs that can activate neutrophil integrin expression. These lipids comprise the majority of DXA_3_ generated by platelets, are formed in ng amounts (24.3±6.1 ng/2×10^8^) and remain membrane bound. Pharmacological studies revealed DXA_3_-PE formation involves cyclooxygenase-1 (COX), protease-activated receptors (PAR) 1 and 4, cytosolic phospholipase A_2_ (cPLA_2_), phospholipase C and intracellular calcium. They are generated primarily via esterification of newly formed DXA_3,_ but can also be formed *in vitro* via co-oxidation of PE during COX-1 co-oxidation of arachidonate. All four DXA_3_-PEs were detected in human clots. Purified platelet DXA_3_-PE activated neutrophil Mac-1 expression, independently of its hydrolysis to the free eicosanoid. This study demonstrates the structures and cellular synthetic pathway for a family of leukocyte-activating platelet phospholipids generated on acute activation, adding to the growing evidence that enzymatic PE oxidation is a physiological event in innate immune cells.

## Highlights

• Four enzymatically oxidized phospholipids are described, termed DXA_3_-PE in platelets.
• DXA_3_-PE forms by COX-1 oxidation of arachidonate, then esterification into PE.
• DXA_3_-PE formation requires calcium, phospholipase C and phospholipase A2.
• DXA_3_-PEs purified from platelets activates neutrophil Mac-1 expression.

## Graphical abstract

**Figure:**
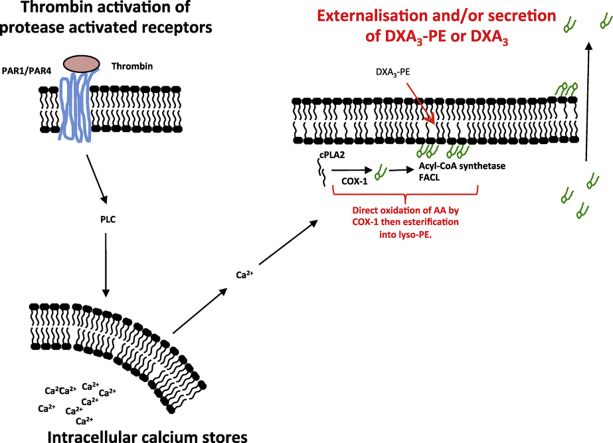

## 1 Introduction

Platelets generate eicosanoids including thromboxane A_2_ (TXA_2_), 12-hydroxyeicosatetraenoic acid (12-HETE), and small amounts of prostaglandins (PGs), PGE_2_, and D_2_. We recently described a new platelet eicosanoid, proposed to be 8-hydroxy-9,11-dioxolane eicosatetraenoic acid (DXA_3_) [1]. Full structural characterization of this lipid remains to be completed once we have sufficient quantities purified from platelets or COX reactions. Importantly, we found that DXA_3_ both primes and activates human neutrophils at nM-μM concentrations [1].

Platelets also form enzymatically-oxidized phospholipids (eoxPL) that contain PGs or HETEs at the *sn2* position [2], [3]. These generally comprise phosphatidylethanolamines (PE) with 16:0p, 18:1p, 18:0p or 18:0a predominating at *sn1*. We recently showed that thrombin-activated platelets generate over 100 diverse eoxPL on acute activation of platelets, including n3 and n6 fatty acids, and both single and multiply oxygenated forms at *sn2* [4]. This indicated that eoxPL formation is a rapid and wide-ranging process of importance to innate immunity. In support, HETE-PEs can modulate a number of relevant events *in vitro* including enhancing clotting factor activities, modulating monocyte cytokine generation and enhancing neutrophil antibacterial actions [3], [5], [6]. Furthermore, two recent studies have shown a critical role for these lipids in mediating ferroptotic cell death [7], [8].

Herein, we describe the detailed cellular and enzymatic biosynthesis pathways for four PE-esterified forms of DXA_3_. A co-ordinated series of enzymes and signaling mediators are required, including the fast esterification of newly formed DXA_3_ free acid. A quantitative assay showed the majority of platelet DXA_3_ generated on thrombin activation is PE-esterified. DXA_3_-PEs remain cell associated, are detected in human clots and activate neutrophil integrin expression, independently of their hydrolysis to the free acid analog. In summary, DXA_3_-PEs are platelet-derived lipids that can activate neutrophils adding to the growing evidence for enzymatic phospholipid oxidation as a physiological process of importance during early innate immunity.

## 2 Materials and methods

### 2.1 Materials

Lipids and lipid standards were purchased from Avanti Polar Lipids (Alabaster, Alabama) or Cayman Chemical (Ann Arbor, Michigan). Deuterated standards are as follows: PGE_2_-*d4*: 9-oxo-11α,15S-dihydroxy-prosta-5Z,13E-dien-1-oic-3,3,4,4-*d*_*4*_ acid, ≥99% deuterated forms. HPLC grade solvents were from Thermo Fisher Scientific (Hemel Hempstead, Hertfordshire UK). PAR-1 and −4 agonists and U73112, U73343 were from Tocris Biosciences (Bristol, UK). COX-1 inhibitor (Sc-560) was from Cayman Chemical. Platelet signaling inhibitors (oleyloxyethylphosphocholine (OOEPC), bromoenol lactone (BEL), cytosolic phospholipase A_2α_ (cPLA_2α_) inhibitor (N-((2S,4R)−4-(Biphenyl-2-ylmethyl-isobutyl-amino)-1-[2-(2,4-difluorobenzoyl)-benzoyl]-pyrrolidin-2-ylmethyl}-3-[4-(2,4-dioxothiazolidin-5-ylidenemethyl)-phenyl]acrylamide, HCl), Gö 6850 and wortmannin were from Calbiochem (UK). All other reagents were from Sigma-Aldrich unless otherwise stated. Ovine COX-1 was from Cayman Chemical or purified as described [9], [10].

### 2.2 Oxidation of phospholipid-esterified arachidonate by purified COX-1

Apo-COX-1 was stored in 80 mM Tris, pH 7.8, at −80 °C. For heme reconstitution, Apo-COX-1 (35 µg) was preincubated on ice for 20 min with 2 M equivalents of hematin in phosphate buffer (100 mM potassium phosphate buffer, pH 7.4). Then, 3.5 μg holo-enzyme was added to 1 ml phosphate buffer with 500 µmol/L phenol and incubated for 3 min at 37 °C in the presence of 150 µM arachidonate (AA), 1-stearoly-2-arachidonyl-PE (SAPE) or both. The reaction was stopped using ice-cold lipid extraction solvent, and immediate extraction of lipids, after addition of 5 ng DMPE internal standard. In some experiments, 10 μM diethylenetriaminepentaacetic acid (DTPA) was added just before holoCOX-1. 18:0a/DXA_3_-PE was analyzed using reverse phase LC-MS/MS as described below.

### 2.3 Isolation of human platelets

Human blood donations were approved by the Cardiff University School of Medicine Ethics Committee and were with informed consent (SMREC 12/37, SMREC 12/10), and according to the Declaration of Helsinki. Exclusion criteria was a known sensitivity to aspirin. For studies on isolated platelets, whole blood was collected from healthy volunteers free from non-steroidal anti-inflammatory drugs for at least 14 days into acid-citrate-dextrose (ACD; 85 mmol/L trisodium citrate, 65 mmol/L citric acid, 100 mmol/L glucose) (blood:ACD, 8.1:1.9, v/v) and centrifuged at 250*g* for 10 min at room temperature. Platelet-rich plasma was collected and centrifuged at 900*g* for 10 min, and the pellet resuspended in Tyrode's buffer (134 mmol/L NaCl, 12 mmol/L NaHCO_3_, 2.9 mmol/L KCl, 0.34 mmol/L Na_2_HPO_4_, 1.0 mmol/L MgCl_2_,10 mmol/L Hepes, 5 mmol/L glucose, pH 7.4) containing ACD (9:1, v/v). Platelets were centrifuged at 800*g* for 10 min then resuspended in Tyrode's buffer at 2×108 ml^−1^. Platelets were activated at 37 °C in the presence of 1 mmol/L CaCl_2_ for varying times, with 0.2 U ml^−1^ thrombin, 10 μg/ml collagen, 10 μmol/L A23187, 20 μmol/L TFLLR-NH_2_, or 150 μmol/L AY-NH_2_ before lipid extraction as below. Experiments involving signaling inhibitors included a 10 min preincubation at room temperature. In some experiments, calcium was omitted from buffers. For separation of cells from microparticles, platelets were centrifuged at 970*g* for 5 min, then supernatants re-spun at 16,060*g* for 5 min. For aspirin supplementation, blood samples were first obtained following a 14-day NSAID-free period for baseline determinations of eicosanoids. Subjects were administered 75 mg/day aspirin for 7 days, then provided a second blood sample. Platelets were isolated and activated *in vitro* using 0.2 U/ml thrombin, as described above, then lipids extracted as described below.

### 2.4 Clot isolation

Blood was allowed to clot for 1 h at 24 °C, and spun at 1730*g* for 10 min. Clot samples were placed in a pre-frozen mortar and pestle on dry ice and further cooled with liquid nitrogen. 250 mg clot was ground up in 3 ml PBS pH 7.4 containing 10 mM DTPA. Lipids were extracted from 1 ml samples using the method below.

### 2.5 Lipid extraction

Lipids were extracted by adding a solvent mixture (1 mol/L acetic acid, isopropyl alcohol, hexane (2:20:30, v/v/v)) to the sample at a ratio of 2.5–1 ml sample, vortexing, and then adding 2.5 ml of hexane [11]. Where quantitation was required, 5–10 ng PGE_2_-d4, and di-14:0-phosphatidylethanolamine (DMPE) were added to samples before extraction, as internal standards. After vortexing and centrifugation, lipids were recovered in the upper hexane layer. The samples were then re-extracted by addition of an equal volume of hexane. The combined hexane layers were dried and analyzed for free or esterified PGs using LC-MS/MS as below.

#### 2.5.1 Reversed phase LC-MS/MS and LC/MS^3^ of esterified DXA_3_

For MRM analysis, lipids were separated on a C_18_ Luna, 3 µm, 150 mm×2 mm column (Phenomenex), using a gradient of 50–100% B over 10 min followed by 30 min at 100% B (Solvent A: methanol:acetonitrile:water, 1 mM ammonium acetate, 60:20:20. Solvent B: methanol, 1 mM ammonium acetate) with a flow rate of 200 μl/min. Esterified DXA_3_ was monitored on a 4000 Q-Trap (Sciex) as precursors of *m/z* 770.6, 796.6, 798.6 and 814.7 fragmenting to product ions at *m/z* 351.2. For high resolution analysis, a reversed-phase UPLC Fourier Transform MS method was used (Thermo Scientific Orbitrap Elite). Analysis was performed using heated electrospray ionization (h-ESI) in negative ion mode at sheet, auxiliary and sweep gas flows of 70, 20, and 0, and capillary and source heater temperatures at 300 and 350 °C, respectively. Data dependent MS^3^ of *m/z* 351 from esterified DXA_3_ was carried out in negative FTMS mode with a resolving power of 15,000. Lipid extracts were separated on a C18 Hypersil Gold, 1.9 µm, 100×2.1 mm column using a gradient (A, methanol/acetonitrile/water containing 1 mM ammonium acetate, at a ratio 60:20:20; B, methanol, 1 mM ammonium acetate) with flow rate 200 μl min ^–1^, starting at 50% B and maintaining for 10 min. The gradient increased to 100% B over 15 min and returning to initial conditions for 5 min. Orbitrap analysis was performed using heated electrospray ionization (h-ESI) in negative ion mode at sheath, auxiliary, and sweep gas flows of 30, 10, and 0, respectively. Capillary and source heater temperatures were 275 and 250 °C, respectively, at 30,000 resolution, in full scan mode. LC/MS of precursor ions were monitored using accurate mass in FTMS mode. Negative MS/MS spectra were acquired using higher energy collision-induced-dissociation (HCD). Data dependent MS^3^ of *m/z* 351 was carried out in ITMS mode on the LTQ Ion Trap. Where fold changes are presented, data are shown as A/IS: analyte divided by internal standard.

#### 2.5.2 Phospholipase A_2_ Hydrolysis of esterified DXA_3_ and quantitation of free acid form

PE was purified from thrombin-activated platelet lipid extracts using normal phase HPLC as follows: extracts resuspended in normal phase solvents (50:50 of solvents A:B (A, hexane: propan-2-ol, 3:2; B, solvent A:water, 94.5:5.5)) were separated on a Spherisorb S5W 4.6×150-mm column (Waters Ltd., Estree, Hertfordshire, UK) using a gradient of 50–100% B over 25 min at a flow rate of 1.5 ml min^−1^ [12]. Absorbance was monitored at 205 nm and products identified by retention time comparison using a mixture of standard phospholipids (bovine brain PC and PE, 25 mg/ml). Fractions were collected at 30 s intervals and analyzed by direct flow injection, for subsequent analysis by ESI/MS/MS. Flow injection analysis of esterified DXA_3_s was performed by injecting 20 μl of each fraction under flow (1 ml min^−1^) in methanol into the electrospray source, with specific MRM transitions monitored using *m/z* 351 as the product ion, on a 4000 Q-trap. PE fractions were dried using N_2_, then resuspended in 1 ml buffer (150 mmol/L NaCl, 5 mmol/L CaCl_2_, 10 mmol/L Tris (Trizma base), pH 8.9). 200 μg snake venom phospholipase A_2_ (PLA_2_) from Sigma-Aldrich was added, and incubated for 60 min at 37 °C. Lipids were re-extracted as above, using hexane:isopropanol:acetic acid. As a purified standard is not yet available, we synthesized and purified a biogenic standard using COX-1 (Hinz et al., in review). Quantitation was achieved using PGE*-d4* as internal standard. For analysis of free DXA_3_, the following HPLC conditions were used: C18 Spherisorb ODS2, 5 µm particle size, 150×4.6 mm (Waters Ltd., Elstree, Hertfordshire, UK) on a Sciex 4000 Q-Trap. The solvent system was 75% water, 25% acetonitrile, 1% glacial acetic acid Solvent A, 60% methanol, 40% acetonitrile, 1% glacial acetic acid at 1 ml min^−1^ Solvent B. Solvent B was increased from 50–90% over 20 min and at 25 min returned to 50% [13].

### 2.6 Isolation of DXA_3_-PEs from platelet lipid extracts

Washed human platelets were activated using A23187 (10 μM, 30 min, 37 °C) with 1 mM CaCl_2_ followed by lipid extraction. DXA_3_-PEs were isolated using a Discovery C18 HPLC column (25 cm×4.6 mm, 5 µm particle size (Sulpeco)) at 1 ml min^−1^, and 50–100% mobile phase B (A: water, 2.5 mM ammonium acetate, B: methanol, 1 mM ammonium acetate) over 15 min, held at 100% B for 20 min. Fractions were collected at 1 min intervals then analyzed using LC-MS/MS. DXA_3_-PE containing fractions were combined and evaporated to dryness using a Rapidvap N2/48 evaporation system (Labconco Corporation), resuspended in methanol, then stored at −80 °C until use.

### 2.7 Isolation and activation of human neutrophils

Human neutrophils were isolated from 20 ml citrate anticoagulated whole blood, and resuspended in Krebs buffer. Briefly, blood was mixed 1:3 with 2% trisodium citrate (wt/vol) and HetaSep (Stemcell technologies) and allowed to sediment for 45 min at 20 °C. The upper plasma layer was recovered and under laid with ice-cold Lymphoprep (2:1 for plasma/Lymphoprep) and centrifuged at 800*g* for 20 min at 4 °C. The pellet was resuspended in ice-cold PBS and 0.4% sodium tricitrate (wt/vol) and centrifuged at 400*g* for 5 min at 4 °C. Contaminating erythrocytes were removed using up to three cycles of hypotonic lysis. Finally, cells were resuspended in a small volume of Krebs buffer (100 mmol/L NaCl, 50 mmol/L HEPES, 5 mmol/L KCl, 1 mmol/L MgCl_2_, 1 mmol/L NaH_2_PO_4_, 1 mmol/L CaCl_2_, and 2 mmol/L D-glucose, pH 7.4), counted and kept on ice. Neutrophils were diluted to 2×106 cells/ml and incubated with or without HPLC purified DXA_3_-PE (equivalent to lipids from 2×108 platelets/ml), vehicle (methanol or DMSO), 1 μM 1-stearoyl-2-arachidonyl-PE (SAPE), or 1 μM fMLP for 20 min at 37 °C. In some experiments, neutrophils were pre-treated with 1.60 µM U-75302 (Cayman Chemicals) or 1 µM LY255283 (Sigma-Aldrich) for 10 min at room temperature. Cells were blocked using 5% mouse serum in PBS (containing 0.5% BSA, 5 mmol/L EDTA and 2 mmol/L sodium azide) for 1 h on ice and centrifuged at 320*g* for 5 min at 4 °C. Anti-human CD11b-Alexa Fluor 647 (0.0625 μg, BioLegend) or isotype control were added and incubated for 30 min on ice. Neutrophils were washed twice with ice-cold PBS (containing 0.5% BSA, 5 mmol/L EDTA and 2 mmol/L sodium azide) and analyzed on a Cyan ADP flow cytometer (Beckman) and identified by forward and side scatter and Alexa Fluor 647.

### 2.8 Statistics

Data on platelets are representative of at least three separate donors, with samples run in triplicate for each experiment. Data are expressed as mean±SEM, of three separate determinations. Statistical significance was assessed using an unpaired, two-tailed Students *t*-test. Where the difference between more than two sets of data was analyzed, one-way ANOVA was used followed by Bonferroni multiple comparisons test, as indicated on legends. p<0.05 was considered statistically significant.

## 3 Results

### 3.1 Human platelets generate phospholipid-esterified DXA_3_ on acute activation

**Fig. 1:**
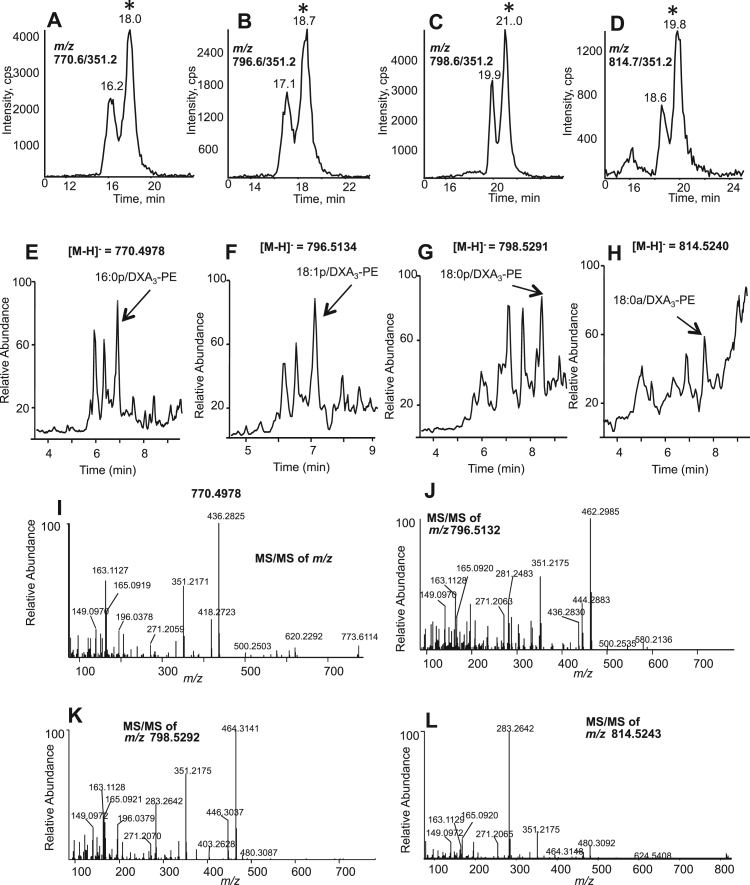
Identification and MS/MS fragmentation of esterified DXA_3_-PE generated by human platelets. *Panels A-D. Lipidomic identification of esterified DXA*_*3*_. Lipid extracts from thrombin-activated platelets (0.2 U/ml, 30 min) were separated using reverse phase LC-MS/MS on a Luna column, monitoring precursor to *m/z* 351.2 for: *Panel A*: 770.6, *Panel B*: 796.6, *Panel C*: 798.6 and *Panel D*: 814.7. The second eluting lipid marked with * was identified as DXA_3_-PE. *Panels E-H. High resolution LC/MS analysis of DXA*_*3*_*-PEs.* Platelet lipid extracts were analyzed using an Orbitrap Elite, with a Hypersil Gold column, in full scan mode at 30,000 resolution. Extraction ion chromatograms corresponding to precursor *m/z* 770.4978, 796.5134, 798.5291 and 814.5240 are shown, with DXA_3_-PE labeled by arrow. *Panels I-L*. *High resolution LC-MS/MS spectra of DXA*_*3*_*-PEs.* Data-dependent LC-MS/MS spectra were acquired using higher energy collision-induced dissociation (HCD) fragmentation, with Orbitrap detection.

**Fig. 2:**
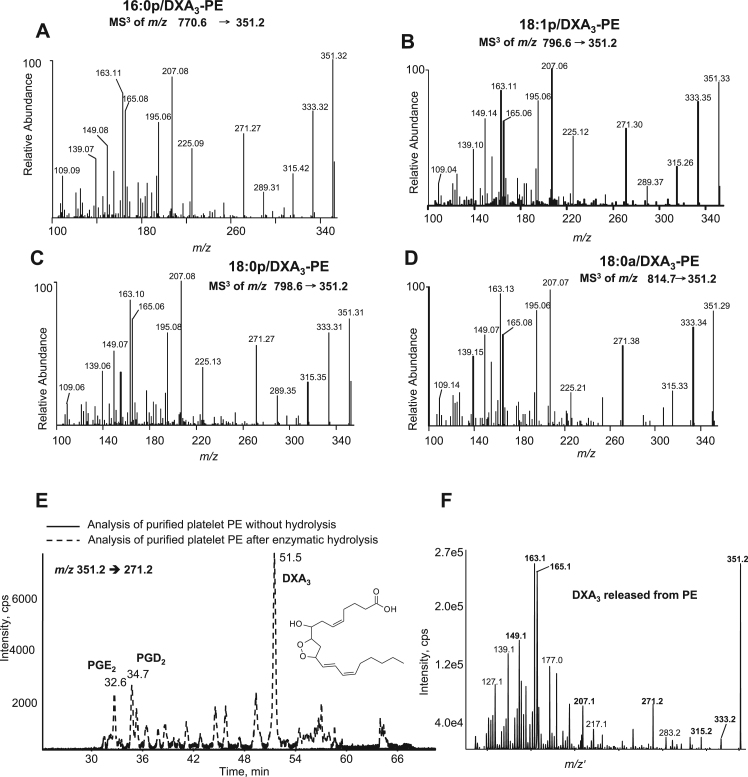
MS^3^ of DXA_3_-PEs, and its hydrolysis from purified PE fractions further confirms structure. *Panels A-D. LC/MS*^*3*^*of DXA*_*3*_*-PE confirms ions that originate from m/z 351.2.* Data dependent LC/MS^3^ spectra were acquired in ITMS mode on the Orbitrap platform using collision-induced dissociation, with precursor to *m/z* 351.2 as product ion. *Panels E. DXA*_*3*_*is released from platelet PE on hydrolysis.* PE was purified from lipids extracted from ionophore activated platelets, then analyzed for free DXA_3_ using LC-MS/MS (*m/z* 351.2-271.2) before (solid line) or after (dashed line) hydrolysis using snake venom PLA_2_, as described in Methods. LC-MS/MS was undertaken on the 4000 Q-Trap, as described for free DXA_3_. *Panel F. MS/MS spectrum confirming the lipid eluting at 51.5 min as DXA*_*3*_. Data dependent LC-MS/MS was undertaken during elution of the lipid at 51.5 min as described in Methods.

Previously we used precursor LC-MS/MS to screen for parents of *m/z* 351.2 in lipid extracts from thrombin-activated platelets, in order to find esterified prostaglandins. This demonstrated several series of ions consistent with four phosphatidylethanolamine (PE) species, namely 16:0p, 18:1p, 18:0p and 18:0a-PE, where p refers to plasmalogen and a to acyl, for the *sn1* fatty acid [2]. Early eluting peaks were identified as esterified PGE_2_/D_2_, but others remained uncharacterised. Herein, a later eluting series are identified as DXA_3_-PEs using MS/MS and MS^3^ both for the esterified forms and following cPLA_2_ hydrolysis of purified platelet PE fractions (Fig. 1, Fig. 2).

Using precursor to product *m/z* 351.2 in multiple reaction monitoring (MRM) mode, two lipids were detected for each PE parent ion (Fig. 1A-D). However, when analyzed using high resolution Orbitrap MS, monitoring exact *m/z*, three lipids were seen (Fig. 1E-H). The first in each MS chromatogram represents co-eluting PGE_2_/D_2_-PEs (Fig. 1E-H) [2]. These are not detected in MS/MS mode since PGE_2_ and D_2_ do not survive as intact carboxylate anions during collision-induced-dissociation of their PE analogs (Fig. 1A-D) [2].

**Fig. 3:**
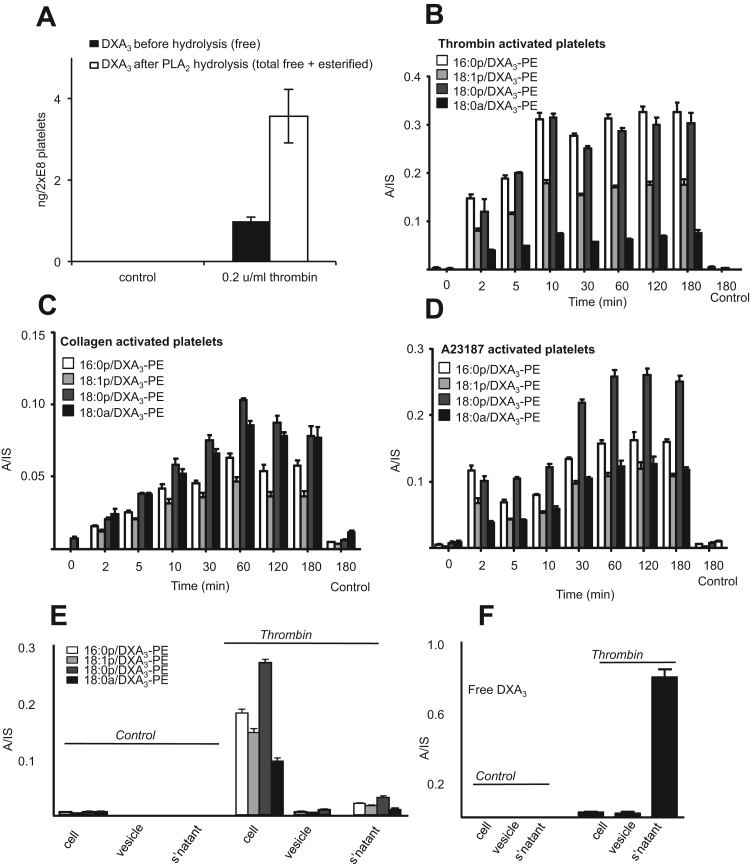
Quantification of DXA_3_ released after hydrolysis of platelet PE and acute generation of DXA_3_-PE by human platelets, which is retained by the cells. *Panel A. Quantification of DXA*_*3*_*released after hydrolysis shows higher levels in PE than free*. Lipid extract of thrombin activated platelets was, hydrolyzed using snake venom PLA_2_ and analyzed for free DXA_3_ using LC-MS/MS before and after hydrolysis. Experiment was repeated on four separate donors, a representative donor is shown (n=3, mean±SEM). *Panels B-D. Generation of PE-esterified DXA*_*3*_*by human platelets.* Washed platelets were activated for varying times, using 0.2 unit.ml^−1^ thrombin, 10 μg/ml collagen, or 10 μmol/L A23187, then lipids extracted and analyzed using reverse-phase LC-MS/MS, monitoring precursor [M-H]^-^ → *m/z* 351.2 as described in Methods. *Panels E,F. Esterified DXA*_*3*_*is retained by the cells while the free acid isomer is released.* Thrombin-activated platelets were pelleted, then supernatant centrifuged at 16,060×*g* for 10 min to pellet microparticles before lipid extraction. DXA_3_-PEs and free DXA_3_ were analyzed using reverse-phase LC-MS/MS, as described in Methods. A/IS: analyte:internal standard.

MS/MS of the third lipid in each series (marked with * in Fig. 1 A-D) demonstrated similar product ions for all four parent PEs, and several were consistent with DXA_3_, notably ions at *m/z* 149, 163 and 165 (Fig. 1I-L). A large product ion at *m/z* 283.2 was also detected for *m/z* 814.5, indicating 18:0a (Fig. 1L). Neutral loss ions at *m/z* corresponding to [M-352]^-^ and [M-334]^-^ were seen in all spectra for loss of DXA_3_ and the DXA_3_ ketene, respectively. MS^3^ of the *m/z* 351.2 ion from all four PEs confirmed the structure of the lipid as DXA_3_ (Fig. 2A-D) [1]. This showed characteristic product ions, including prominent ones at *m/z* 163, 165, and also 207, 225 and 271. To further prove DXA_3_ was attached to PE, the lipids were purified using HPLC, then saponified using snake venom PLA_2_, as described in Materials and Methods. Analysis of saponified PE using the *m/z* 351.2 → 271.2 showed some PGE_2_ and D_2_ at 32.6 and 34.7 min respectively, however DXA_3_ was seen at 51.5 min, with MS/MS confirming its structure (Fig. 2E,F). To quantify DXA_3_ attached to PE, free DXA_3_ in lipid extracts derived from thrombin-activated platelets was quantified before and after hydrolysis using snake venom PLA_2_ (Fig. 3A). Total esterified DXA_3_ in platelets from four donors was determined to be 24.3±6.1 out of 31.6±8.1 ng /2×108 platelets/ml (mean±SEM). This indicates that the majority of DXA_3_ is esterified, with structural data confirming the four lipids as 16:0p/DXA_3_-PE, 18:1p/DXA_3_-PE, 18:0p/DXA_3_-PE and 18:0a/DXA_3_-PE.

### 3.2 Characterization of platelet signaling pathways involved in DXA_3_-PE generation

**Fig. 4:**
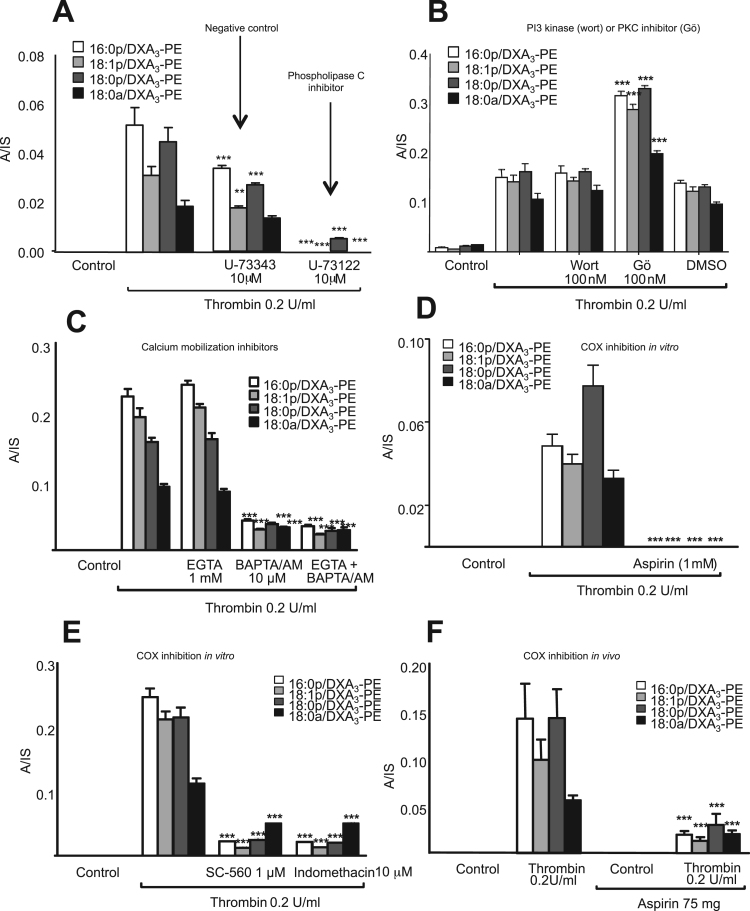
DXA_3_-PE formation requires the activity of PLC, calcium, cPLA_2_ and COX-1, and occurs via esterification of free DXA_3._*Panels A-C Effects of signaling inhibitors on DXA*_*3*_*formation.* Platelets were incubated with inhibitors 10 min prior to thrombin activation (0.2 U/ml for 30 min at 37 °C). Lipids were extracted and analyzed using LC-MS/MS monitoring precursor [M-H]^-^*m/z* 351.2 → 165.1, as described in Methods. Data are representative of experiments repeated at least three times on different donors (n=3, mean±SEM). *** P<0.001 versus thrombin, using ANOVA and Bonferroni Post Hoc Test. *Panel A.* U-73112, 10 µM (PLC), or its negative control U-73343. *Panel B.* wortmannin, 100 nM (PI3 kinase), Gö 6850 (protein kinase C), 100 nM (PKC) or vehicle (DMSO, 0.5%), *Panel C*. EGTA 1 mM (extracellular Ca^2+^) or BAPTA-AM 10 mM (intracellular Ca^2+^). *Panels D,E. Generation of DXA*_*3*_*-PE is sensitive to COX inhibition in vitro.* Platelets were incubated with 1 mM aspirin, 1 μM SC-560 or 10 μM indomethacin prior to thrombin activation (0.2 U/ml for 30 min at 37 °C). Lipids were extracted and analyzed as described in Methods. Levels are expressed as ratio analyte to internal standard. Data are representative of experiments repeated at least three times on different donors (n=3, mean±SEM). *Panel F. In vivo aspirin supplementation blocks generation of DXA*_*3*_*-PE.* Lipids were analyzed following thrombin activation of washed platelets, before or after supplementation with 75 mg/day aspirin for 7 days. Data are representative of five independent donors (n=5, mean±SEM); ***p<0.001 versus thrombin alone, using ANOVA and Bonferroni Post Hoc Test. Levels of DXA_3_-PEs are expressed as ratio analyte to internal standard. A/IS: analyte:internal standard.

**Fig. 5:**
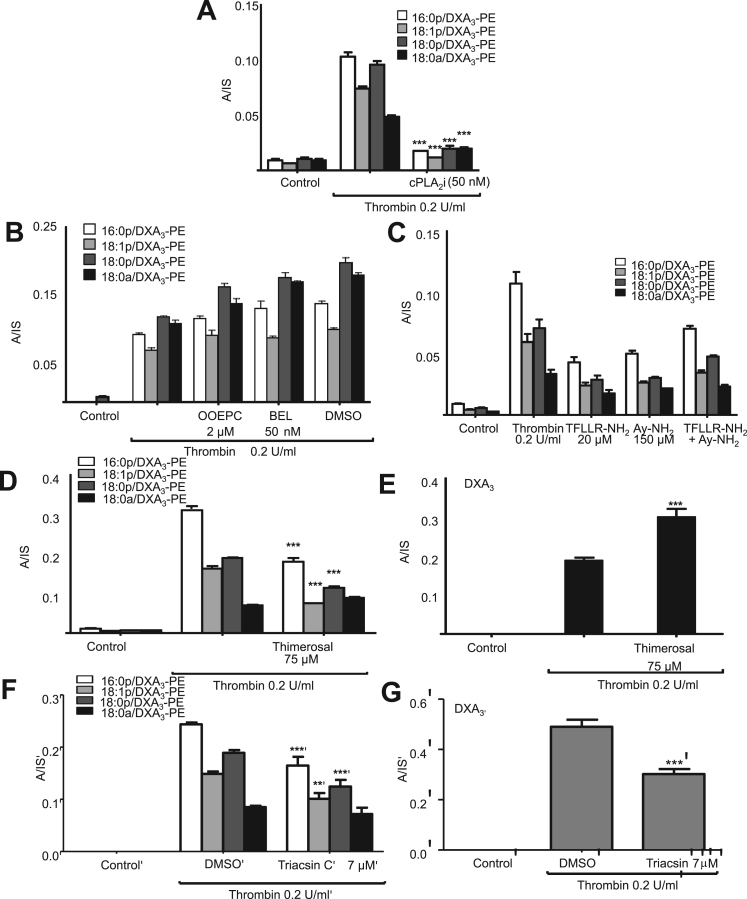
DXA_3_-PE generation requires cPLA_2_, PAR receptors and LPAT but not other PLA_2_ isoforms. *Panel A. Inhibition of DXA*_*3*_*-PE generation by cPLA*_*2*_*blockade*. Platelets were incubated with cPLA_2_i 50 nM (cPLA_2_ inhibitor) prior to thrombin activation (0.2 U/ml for 30 min at 37 °C). Lipids were extracted and analyzed as described in Methods. Levels are expressed as ratio analyte to internal standard. Data are representative of experiments repeated at least three times on different donors (n=3, mean±SEM). *Panel B. Formation of DXA*_*3*_*-PE does not require iPLA*_*2*_*or sPLA*_*2*_. DXA_3_-PE formation by platelets incubated with 2 µM OOEPC (sPLA_2_), 50 nM BEL (iPLA_2_) or vehicle (DMSO, 0.5%) before thrombin activation was determined. Levels are expressed as analyte:internal standard. *Panel C. DXA*_*3*_*-PE is generated via PAR-1 and PAR-4 receptor stimulation.* Washed platelets were activated with a PAR-1 agonist, TFLLR-NH_2_ (20 μM), and/or a PAR-4 agonist, AY-NH_2_ (150 μM), for 30 min at 37 °C then analyzed, as described in Methods. *Panels D,E. Generation of DXA*_*3*_*-PE is inhibited by thimerosal, while free DXA*_*3*_*is enhanced.* Washed platelets were incubated with 75 µM of thimerosal for 30 min at 37 °C prior to thrombin activation, before lipid extraction and analysis. *Panels F,G. Generation of free and esterified DXA*_*3*_*-PE is inhibited by triascin C.* Washed platelets were incubated with 7 µM of triascin C for 30 min at 37 °C prior to thrombin activation, before lipid extraction and analysis. Levels are expressed as analyte:internal standard. Data are representative of experiments repeated at least three times on different donors (n=3, mean±SEM). ***p<0.001 versus thrombin, using ANOVA and Bonferroni Post Hoc Test. A/IS: analyte:internal standard.

On platelet activation, DXA_3_-PE was generated early, in particular in response to thrombin where it plateaued after 10 min (Fig. 3B-D). DXA_3_-PE was retained by the cells, in contrast to free DXA_3_, which is primarily secreted, indicating that the two forms partition differently (Fig. 3E,F). DXA_3_-PE formation required PLC, but not PI3 kinase, while inhibition of PKC significantly increased DXA_3_-PE generation (Fig. 4A,B). The negative control (U-73373) for the PLC inhibitor (U-73112) showed approximately 50% inhibition, but far less than seen using U-73112 (Fig. 4A). This suggests some off target effects of U-73112, but supports PLC involvement in DXA_3_-PE generation. Formation of DXA_3_-PEs was dependent on intracellular but not extracellular calcium (Fig. 4C). An essential role for COX-1 was revealed through either *in vivo* (7 days of 75 mg/day aspirin) or *in vitro* inhibition (SC-560, aspirin, indomethacin) inhibition (Fig. 4D-F), while cPLA_2_ but not iPLA_2_ or sPLA_2_ was also required (Fig. 5A,B). Last, formation of DXA_3_-PE was triggered *via* PAR-1 and −4 receptors (Fig. 5C). Notably, the timescale for formation of esterified DXA_3_ is very similar to that of the free acid form with a fast generation within the first 10 min of activation [1].

### 3.3 Enzymatic mechanism of DXA_3_-PE formation by COX-1

**Fig. 6:**
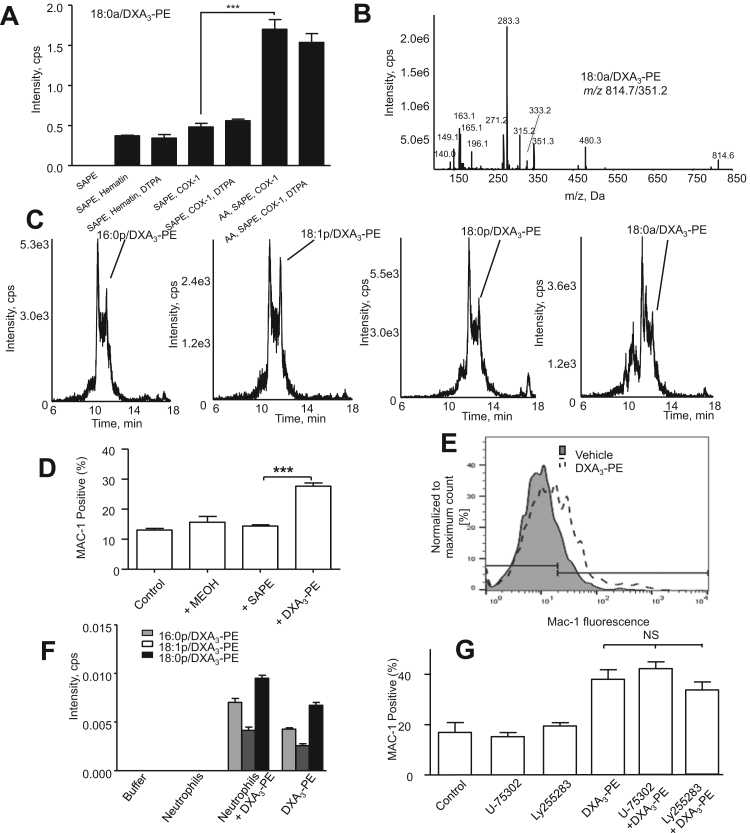
Characterization of DXA_3_-PE formation by purified COX-1, formation of DXA_3_-PE in clots and activation of neutrophils using semi-purified DXA_3_-PE. *Panels A,B. COX-1 doesn’t directly oxidize PE.* 3.5 μg of holoCOX-1 was incubated with either 150 µM of AA, 150 µM of SAPE or liposomes containing AA and SAPE, for 3 min at 37 °C, in the presence or absence of 10 μM DTPA. Lipids were extracted and analyzed by reverse-phase LC-MS/MS, monitoring precursor [M-H]^-^ → *m/z* 351.2. Levels of 18:0a/DxA_3_-PE are expressed as ratio of analyte to internal standard/3.5 μg enzyme generated over 3 min (n=3, mean±SEM). Data are representative of ≥3 separate experiments. ***p ˂ 0.001 using ANOVA and Bonferroni Post Hoc Test. *Panel C. LC-MS/MS of DXA*_*3*_*-PE formed in vitro via COX-1 during AA co-oxidation.* Lipid extracts were separated using reverse-phase LC/MS, monitoring [M-H]^-^ → *m/z* 351.2, with spectra taken at the peak of elution at 20.3 min *Panel C. Detection of four DXA*_*3*_*-PE isomers in human clots.* Clot lipids were extracted and analyzed as described in Methods. Representative LC-MS/MS data from one of three different donors are shown. Note retention time is earlier since these were analyzed using a different LC-MS/MS platform (Sciex 6500 Q-Trap, although with identical LC conditions to Fig. 1 A-D). *Panels D.E. DXA*_*3*_*-PE lipid fraction activates neutrophil integrin expression*. Human neutrophils were isolated and activated using either fMLP or a platelet-derived DXA_3_-PE-containing lipid isolate, as described in Methods. Mac-1 expression was determined using CD11b-Alexa Flour 647 detected by flow cytometry. *Panel D. DXA*_*3*_*-PE, but not SAPE (the unoxidized control lipid), activates Mac-1 expression.* Neutrophils were incubated with/without DXA_3_-PE or 1 mM SAPE as described in Methods. Mac-1 expression was determined using CD11b-Alexa Fluor 647 detected by flow cytometry, n=3, mean±SEM. *Panel E.* A representative histogram is shown. *Panel F. Neutrophils do not hydrolyze DXA*_*3*_*-PE*. Neutrophils (2×106/ml) were incubated with DXA_3_-PE for 30 min at 37°, before lipid extraction and analysis using LC/MS/MS as described in Methods. Free DXA_3_ was not detected under these conditions. *Panel G. DXA*_*3*_*-PE activation of Mac-1 is independent of BLT1 and BLT2.* Neutrophils were incubated with/without DXA_3_-PE as described in Methods, with pretreatment (10 min, 22 °C) with 1.6 mM U-75302 (BLT1 antagonist) or 1 mM LY255283 (BLT2 antagonist). Mac-1 expression was determined using CD11b-Alexa Fluor 647 detected by flow cytometry, n=3, mean±SEM, ANOVA with Bonferroni Post Hoc Test. *** p<0.001.

DXA_3_-PE could form via direct PE oxidation or via oxidation of AA released by cPLA_2_ followed by esterification into PE. The latter is more likely since COX-1 is unable to directly oxidize complex lipids, and DXA_3_-PE formation was sensitive to cPLA_2_ inhibition. In support, thimerosal, an inhibitor of lysophospholipid acyl transferases (LPAT) elevated free DXA_3_, while suppressing DXA_3_-PE formation (Fig. 5D,E). This supports a mechanism whereby AA is hydrolyzed by cPLA_2_, oxidized by COX-1 to DXA_3_, then re-esterified into PE via LPAT enzymes. Triascin C, an inhibitor of fatty acyl Co-A ligase (FACL), inhibited formation of DXA_3_-PE, but also partially blocked free DXA_3_ formation (Fig. 5F,G). COX-1 is generally considered unable to oxidize complex substrates. To confirm this, we incubated 18:0a/20:4-PE with COX-1. Hematin controls showed a small oxidation to form DXA_3_-PE, but this was not increased by inclusion of COX-1 (Fig. 6A,B). However, when AA was added, allowing COX-1 turnover, additional DXA_3_-PE was formed. As this was not sensitive to metal chelation, it likely results from secondary oxidation directly mediated by lipid radicals that escape the active site of COX-1 during turnover (Fig. 6A,B). We note that thimerosal blocked approx. 50% of the generation of DXA_3_-PEs (Fig. 5D). Thus, at least half of these lipids form in platelets via esterification, rather than radical-mediated oxidation.

### 3.4 DXA_3_-PE from human platelets activates human neutrophil integrin expression

DXA_3_-PE was isolated from activated platelet lipid extracts using HPLC. When added at concentrations approximating a physiological platelet concentration (equivalent to 2×108 cells/ml), neutrophil Mac-1 expression increased significantly (Fig. 6D,E). In contrast, the unoxidized analog, 1-stearoyl-2-arachidonyl-PE (SAPE) did not activate Mac-1 (Fig. 6D). When incubated for 30 min with neutrophils, no loss of DXA_3_-PE was seen, nor was free DXA_3_ generated (Fig. 6F). This indicates that hydrolysis of DXA_3_-PE by neutrophil phospholipases did not occur and indicating that Mac-1 activation is stimulated directly by the esterified form. Instead, levels of DXA_3_-PE appeared to slightly increase, likely a matrix effect on extraction from the presence of neutrophil lipids and proteins. Last, to explore the signaling mechanisms, antagonists of leukotriene B4 receptors, BLT1 and BLT2 were included. However, neither prevented the expression of Mac-1 indicating that DXA_3_-PE activates neutrophils by BLT receptor-independent mechanisms (Fig. 6G).

## 4 Discussion

Recent studies identified a platelet lipid, DXA_3_ that forms via enzymatic oxidation of AA by COX-1 in response to physiological agonist activation. This lipid primes and activates human neutrophils at nM-μM concentrations *in vitro* suggesting its likely importance in innate immune responses [1]. In that study, only the free acid lipid was described. Herein, we demonstrate that the majority of DXA_3_ is initially formed esterified to four PEs, remains cell-associated and is generated in human clots *in vitro*. Like the free acid analog, DXA_3_-PE also activates neutrophils at physiologically-relevant concentrations. The study adds to the growing evidence that enzymatic phospholipid oxidation by platelets is a physiological process that occurs during blood clotting and hemostasis and modulates innate immunity [3].

DXA_3_-PE is formed on a similar timescale, within 2–10 min post agonist activation, as free DXA_3_ (Fig. 3 B-D) and entirely from endogenous substrate mobilized during physiological platelet activation [1]. This indicates that a controlled enzymatic formation, similar to the generation of HETE-PEs by platelet 12-lipoxygenase (LOX), macrophage 12/15-LOX and neutrophil 5-LOX. Pharmacological studies indicated involvement of PLC, intracellular calcium, cPLA_2_ and COX-1 [3], [5], [11]. These signaling mediators are also required for free DXA_3_ formation demonstrating that PE-esterified forms require first the generation of the free acid form [1]. This was further supported by observations that an inhibitor of LPAT significantly blocked their formation (Fig. 5D,E). Activation by PAR agonists suggests the involvement of these receptors in thrombin activation, however thrombin can also activate additional receptors, including integrins. Platelets express several PKC isoforms, of which some promote and others inhibit platelet activation. For example, PKCα can be pro- while PKCδ can be anti-aggregatory [14]. Go6850 (at 100 nM) will inhibit a number of PKC isoforms, including α,β,δ and ε [15]. We found that this enhanced generation of DXA_3_-PE suggesting that overall, PKC isoforms are suppressing this pathway. Using purified COX-1, we showed that COX-1 does not directly oxidize 18:0a/20:4-PE (Fig. 6A). However, a small amount of co-oxidation could be mediated during AA oxidation. This suggests an additional route to DXA_3_-PE formation and could account for the thimerosal-insensitive generation seen in platelets (Fig. 5D), however it is likely that non-enzymatic routes to synthesis would be tightly controlled and minimized in platelets via the action of glutathione peroxidases.

The majority of DXA_3_ is detected as esterified to PE in platelets (Fig. 3A). This contrasts with HETE-PEs of which only 30% is attached to phospholipids, and TXB_2_ which has not been found to occur in esterified forms, and suggests that esterification pathways may favor certain oxidized lipids, but this remains to be fully investigated [3]. Recently, we used lipidomics to profile oxidized PL generated by human platelets and found over 100 species all generated on acute activation with thrombin [4]. One family, HETE-PEs, can translocate to the outside of the plasma membrane and is prothrombotic *in vitro*, raising the possibility that membrane PL oxidation is a general phenomenon that regulates innate immune events during acute injury [3].

Herein, DXA_3_-PE was found to activate neutrophil Mac-1 expression, independently of hydrolysis to free DXA_3_ (Fig. 6D-H). This indicates that DXA_3_ could potentially act either as a soluble mediator when free, or alternatively when remaining cell-associated, if facing out of the membrane surface and attached to PE (e.g. during platelet-neutrophil interactions). This would be a new concept for eicosanoid signaling, since traditionally these were only thought of as soluble signaling agents. Previous, eicosanoids were not considered to be re-esterified into phospholipids in significant amounts. This study showing DXA_3_ esterification strengthens previous reports of formation of PGE_2_- and HETE-PE formation as a regulated biological process [2], [3], [5], [6], [11], [16].

We suspected that DXA_3_-PE might activate LTB_4_ receptors, either BLT1 or BLT2, since both are present in neutrophils and known to upregulate Mac-1 expression [17], [18]. However, both were excluded using receptor antagonists (Fig. 6H). Neutrophils express many cell surface receptors, including other G protein coupled receptors, Toll-like receptors and integrins [17]. Thus DXA_3_-PE likely activates other pathways to upregulate Mac-1 expression and further studies are required to delineate this. Indeed whether the free and esterified forms of this lipid can also stimulate additional neutrophil activities including calcium mobilization, superoxide generation and degranulation, also will be determined.

Neutrophil phospholipids that have incorporated exogenous eicosanoids can be subsequently hydrolyzed by secondary cell stimulation, for example releasing free acid 15-HETE [19]. Thus, during blood clotting, phospholipases such as sPLA_2_ could metabolise DXA_3_-PEs from activated platelets generating the free acid analog and thus further supporting innate immune responses to acute injury via soluble diffusion, with this lipid able to signal either in free or esterified forms. In summary, DXA_3_-PEs represent new platelet lipids generated through receptor-regulated cell signaling pathways. The full structural characterization of DXA_3_ and generation of synthetic standards will enable further study of its functions in innate immunity.

## Authorship contributions

CH, MA, HP, MH, VH, SC, DS conducted experiments. CH, MA, VOD, RCM, PWC designed experiments. LJM, CP and SGO provided reagents/clinical samples. MA and VOD wrote the paper. All authors edited the paper.

## Conflicts of interest

The authors declare no competing financial interests.
